# Putative Role of Circulating Human Papillomavirus DNA in the Development of Primary Squamous Cell Carcinoma of the Middle Rectum: A Case Report

**DOI:** 10.3389/fonc.2019.00093

**Published:** 2019-02-21

**Authors:** Maria Raffaella Ambrosio, Remo Vernillo, Sabrina De Carolis, Antonietta Carducci, Lucia Mundo, Alessandro Ginori, Bruno Jim Rocca, Valerio Nardone, Alessandra Lucenti Fei, Tommaso Carfagno, Stefano Lazzi, Monica Cricca, Piero Tosi

**Affiliations:** ^1^Department of Medical Biotechnology, University of Siena, Siena, Italy; ^2^Department of Medical Sciences, Surgery and Neuroscience, University of Siena, Siena, Italy; ^3^Center of Applied Biomedical Research (CRBA), S. Orsola-Malpighi Hospital, Bologna, Italy; ^4^Department of Experimental, Diagnostic and Specialty Medicine, University of Bologna, Bologna, Italy; ^5^Pathology Unit, Ospedale Civico di Carrara, Carrara, Italy; ^6^Retired, Siena, Italy

**Keywords:** circulating HPV, immune evasion, exosomes, middle rectum, cancer

## Abstract

Here we present the case of a patient affected by rectal squamous cell carcinoma in which we demonstrated the presence of Human Papillomavirus (HPV) by a variety of techniques. Collectively, the virus was detected not only in the tumor but also in some regional lymph nodes and in non-neoplastic mucosa of the upper tract of large bowel. By contrast, it was not identifiable in its common sites of entry, namely oral and ano-genital region. We also found HPV DNA in the plasma-derived exosome. Next, by *in vitro* studies, we confirmed the capability of HPV DNA-positive exosomes, isolated from the supernatant of a HPV DNA positive cell line (CaSki), to transfer its DNA to human colon cancer and normal cell lines. In the stroma nearby the tumor mass we were able to demonstrate the presence of virus DNA in the stromal compartment, supporting its potential to be transferred from epithelial cells to the stromal ones. Thus, this case report favors the notion that human papillomavirus DNA can be vehiculated by exosomes in the blood of neoplastic patients and that it can be transferred, at least *in vitro*, to normal and neoplastic cells. Furthermore, we showed the presence of viral DNA and RNA in pluripotent stem cells of non-tumor tissue, suggesting that after viral integration (as demonstrated by p16 and RNA *in situ* hybridization positivity), stem cells might have been activated into cancer stem cells inducing neoplastic transformation of normal tissue through the inactivation of p53, p21, and Rb. It is conceivable that the virus has elicited its oncogenic effect in this specific site and not elsewhere, despite its wide anatomical distribution in the patient, for a local condition of immune suppression, as demonstrated by the increase of T-regulatory (CD4/CD25/FOXP3 positive) and T-exhausted (CD8/PD-1positive) lymphocytes and the M2 polarization (high CD163/CD68 ratio) of macrophages in the neoplastic microenvironment. It is noteworthy that our findings depicted a static picture of a long-lasting dynamic process that might evolve in the development of tumors in other anatomical sites.

## Introduction

Human papillomavirus (HPV) DNA has been detected in a variety of cancer, including colorectal cancer (CRC) (1–4). Through the integration of viral DNA into the human genome, expression of oncoproteins, and inactivation of tumor suppression genes, HPV damages cell proliferation in its site of infection, inducing oncogenesis (2). Recently, HPV DNA has been found as circulating DNA in the serum of patients with HPV-associated invasive carcinoma (5) and its presence has been considered as a useful biomarker for disease recurrence (6). Tumor-derived exosomes and extracellular vesicles are garnering increasing attention because of their ability to transfer bioactive molecules (mRNAs, microRNAs, DNA, and proteins) between neighboring cancerous or normal cells, and to contribute to human cancer progression. Moreover, exosomes and extracellular vesicles have been implicated in HPV transmission and carcinogenesis (7), and the presence of HPV DNA in the serum derived extracellular vesicles (EVs) of patients with breast diseases has been recently reported (8).

While there is a strong association between HPV and squamous cell carcinoma of some sites (e.g., genitals, anus, and head and neck region), the putative role of the virus in colorectal carcinogenesis still remains controversial (9). Detection of HPV in colonic tissue has led to a wide range of studies, analyzing the possibility of a casual or pathogenetic association between HPV and CRC. However, these studies applied different techniques for the detection of the virus, with discordant findings and inconsistencies in result reproducibility (10). In fact, it is very difficult that a causative role of HPV in a cancer can be explained only studying the molecular mechanisms. Since it is a very common virus, its presence can be occasional and the behave of the virus opportunistic. The proof of causation needs both studies on the molecular mechanisms and studies establishing that the probability a tumor occurs is higher when the HPV is present that when it is not, i.e., case control and cohort studies.

CRC is the third most common cause of cancer-related death in the world (11). Among its histotypes, pure squamous cell cancer (SCC) is very unusual, ranging from 0.1 to 0.25 *per* 1,000 CRC; its diagnosis is based on the restrictive criteria given by Williams et al. (12). Risk factors include proctitis, past history of radiotherapy to pelvic region, Schistosomiasis and Amoebiasis infection (13). The exact pathogenic mechanism remains speculative and several theories have been proposed (14, 15).

Here we present the case of a patient affected by rectal SCC in which we demonstrated HPV by immunohistochemistry, polymerase chain reaction (PCR), chromogenic *in situ* hybridization (CISH), and RNA *in situ* hybridization (ISH) for *E6/E7* viral oncogenes mRNA in neoplastic and non-neoplastic tissues, including lymph nodes and sites far from the rectum. We also found HPV DNA in the plasma-derived from the patient, and by *in vitro* studies, we confirmed the capability of HPV DNA positive exosomes to transfer its DNA to recipient cells.

## Background

### Clinical Presentation

A 53 years-old caucasian man presented to our hospital complaining of sub-occlusive syndrome that lasted for 1 week. Family and medical history was unremarkable. Physical examination including digital rectal examination was negative. Colonoscopy showed a 5.0 × 4.5 × 4 cm lesion, 10 cm from the anal verge. The lesion was ulcerated and bleeding, it narrows the lumen and renders difficult to be overcome. Computed tomography (CT)-scan identified a heteroplastic and locally advanced lesion with enlarged lymph nodes (the greatest measuring 13 mm in maximum diameter) along the sigmoid chain. Neither pulmonary, hepatic, splenic localizations nor pleural or peritoneal effusions were identified. An anterior resection of the rectum and regional lymph nodes was performed without colostomy. Postoperatively, the patient underwent adjuvant radiotherapy of 45 Gy delivered to the pelvis in 25 fractions over 5 weeks. To date, after 60 months follow-up, the patient is alive and in good conditions, with no evidence of recurrence. Ethics approval for this study was obtained from the Institutional Review Board at the University of Siena (Italy) and written informed consent was obtained from the patient for the publication of this case report and any potentially-identifying information/images.

### Materials and Methods

Tables 1–3 summarize the main techniques applied in the different samples.

**Table 1:** Synoptical table of the methods applied in the different samples and the respective results obtained.

**Tissue**	**CISH** **HPV**	**RNA scope**	**PCR MY09/11-E6-E1-L1 genes**	**Digital PCR**	**p63** **CK20**	**CK7** **CK5/6**	**p16**	**p53** **p21** **Rb**	**CISH-stem cell marker (OCT3/4) double staining**	**CISH-neuroendocrine marker (chromogranin/synaptophisin) double staining**	**T-reg**	**T-exh**	**M2** **TAM**	**PD-1**	**PDL-1** **PDL-2**
Tumor mass	+	+	+	n.a.	+	–	+	–	–	–	↑	↑	+	+ B	+ T
Surgical margins	+	+	+	n.a.	n.a.	n.a.	–	n.a.	+	–	↓	↓	–	–	–
Tumor–free normal rectal mucosa of the surgical specimen	+	+	+	n.a.	n.a.	n.a.	–	n.a.	+	–	↓	↓	–	–	–
Lymph node of the surgical specimen	–	–	+	n.a.	n.a.	n.a.	+	n.a.	n.a.	n.a.	n.a.	n.a.	n.a.	n.a.	n.a.
Follow up biopsies of caecum/ascending and descending colon	–	–	+	n.a.	n.a.	n.a.	–	n.a.	–	–	↓	↓	–	–	–
Oral swab	n.a.	n.a.	–	n.a.	n.a.	n.a.	n.a	n.a.	n.a.	n.a.	n.a.	n.a.	n.a.	n.a.	n.a.
Penile scrape	n.a.	n.a.	–	n.a.	n.a.	n.a.	n.a	n.a.	n.a.	n.a.	n.a.	n.a.	n.a.	n.a.	n.a.
Plasma-derived Exosomes	n.a.	n.a.	+	n.a.	n.a.	n.a.	n.a	n.a.	n.a.	n.a.	n.a.	n.a.	n.a.	n.a.	n.a.
*In vitro* transfected cells	n.a.	n.a.	+	+	n.a.	n.a.	n.a.	n.a.	n.a.	n.a.	n.a.	n.a.	n.a.	n.a.	n.a.

*+,positive; –, negative; n.a., not assessed; T-reg, T-regulatory lymphocytes (CD25/CD4/FOXP3 positivity); T-exh, T-exhausted lymphocytes (CD8/PD1 positivity); TAM, tumor-associated macrophages (CD163/CD68 ratio); + B, positive in the background; + T, positive in the tumor cells; ↑, increased; ↓, decreased; CISH, chromogenic in situ hybridization; PCR, polymerase chain reaction*.

*p63, CK20, CK7, CK5/6, p53, p21, Rb, OCT3/4, chromogranin, synaptophisin, CD4, CD8, CD25, FOXP3, PDL-1, PDL-2, PD-1, CD68, and CD163 were evaluated by immunohistochemistry*.

**Table 2:** HPV DNA detection with different primer set.

**Primer set**	**Target**	**Forward (5′–3′)**	**Reverse (5′–3′)**	**Length**	**NT position**
E1	HPV16	ATCGTAATWSAGCCWCCAAAATT	TTATCAWATGCCCAYTGTACCAT	188	1,777–1,964
E6	HPV16	AAAGCCACTGTGTCCTGAAGA	CTGGGTTTCTCTACGTGTTCT-	130	424–553
L1	HPV16	TTTGTTACTGTGGTAGATACTAC	GAAAAATAAACTGTAAATCATATTC	140	6,625–6,768

*The nucleotide position (NT) was assessed on the reference sequence NC_001526 (Human Papillomavirus 16 type)*.

**Table 3:** Immunohistochemical analysis.

**Target**	**Clone**	**Source**	**Retrieval/dilution**
p63	4A4	Ventana (Roche)	RTU
CK20	SP33	Ventana (Roche)	RTU
CK5/6	D516B4	Ventana (Roche)	RTU
CK7	SP52	Ventana (Roche)	RTU
p16	P16	Ventana (Roche)	RTU
Synaptophisin	MRQ40	Ventana (Roche)	RTU
Chromogranin	LK2H10	Ventana (Roche)	RTU
OCT3/4	MRQ/10	Ventana (Roche)	RTU
p53	DO/7	Ventana (Roche)	RTU
p21	EPR18021	Abcam	1:150
Rb	Rb1 1F8	Abcam	1:200
PDL-1	SP263	Ventana (Roche)	RTU
PDL-2	366C-9E-5	Biox Cell	RTU
PD-1	NAT105	Ventana (Roche)	RTU
CD25	IL2R.1	ThermoFisher	1:200
CD4	SP35	Ventana (Roche)	RTU
CD8	SP57	Ventana (Roche)	RTU
FOXP3	SP97	Spring	1:50
CD68	KP/1	Ventana (Roche)	RTU
CD163	CD163	Leica Biosystem	1:200

*RTU, ready to use; Ventana- Roche, Mannheim (Germany); Abcam, Cambridge (United Kingdom); Biox Cell, West Lebanon (NH, USA); ThermoFisher, Rockford (USA); Spring, Pleasanton (USA); Leica Biosystem, New Castle (United Kingdom)*.

## Results

### Pathological Characterization

**Figure 1:**
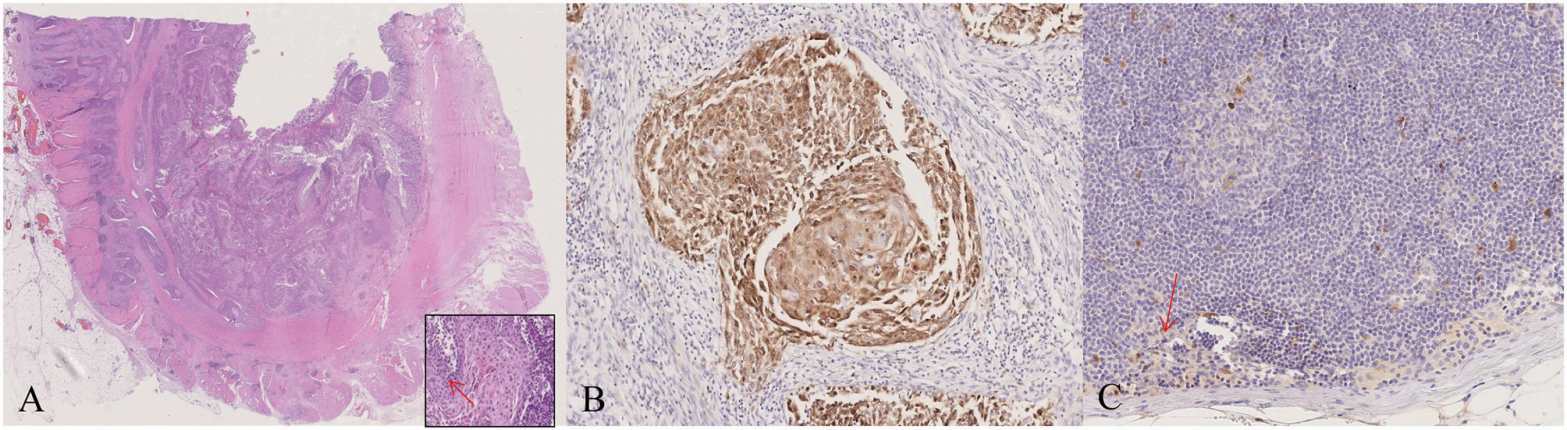
Pathological characterization. The morphological and immunohistochemical evaluation of the surgical specimen showed the presence of a squamous cell carcinoma with basaloid features and koilocyte-like cells (arrow) (**A**, inset), and p16 expression **(B)**; the largest lymph node examined demonstrated p16 positivity in scattered large cells of the germinal center and the subcapsular sinus (arrow) **(C)**. A, **(A)**-inset: haematoxylin and eosin (H&E); **B,C**: p16 staining. Original magnification (O.M.): A, 2.5x; **(A)**-inset, **(B,C)** 20x.

Grossly, an exophytic ulcerative mass with predominantly intraluminal growth and narrowing of the lumen was identified. The lesion measured 5.0 × 4.0 × 3.5 cm and extended throughout the muscular layer but without mesorectal involvement in the extraperitoneal tract of the specimen. On cut section, the mass had a relatively homogeneous appearance with focal areas of necrosis and hemorrhages. Microscopic examination demonstrated a moderately differentiated squamous cell carcinoma with basaloid features and koilocyte-like cells (Figure 1A, inset). Neither perineural nor lymphovascular invasion was present. Surgical margins as well as the regional lymph nodes examined showed no cancer infiltration. The final diagnosis according to Williams et al. criteria was G2 (12), pT3N0Mx, IIA (TNM 2017) primary squamous cell carcinoma of the rectum. By immunohistochemistry (IHC), the neoplastic cells were p63 and CK20 positive, and CK5/6 and CK7 negative, thus ruling out a possible rectal extension from an occult anal neoplasm.

### HPV Detection and Oncogenicity

The atypical localization of a SCC in the rectum and the detection of koilocyte-like differentiated cells within the tumor were indicators of the possible presence of HPV. Therefore, we searched the virus in the tumor mass, in its margins, in tumor-free normal rectal mucosa proximal to the tumor and in the lymph nodes by IHC for p16, PCR assays, CISH for HPV nucleic acids (ZytoFast PLUS CISH Implementation Kit HRP-DAB and ZytoFast HPV type 16/18 Probe, ZytoVision, Bio-Optica, Milan, Italy) and RNA ISH for *E6/E7* mRNA transcripts (RNAscope®2.5 HD Assay- RED, probe HPV 16, Acdbio, Milan, Italy) (Table 1).

By IHC for p16, a useful surrogate biomarker of HPV integration and E7 over-expression (16), strong positivity was detected only in the neoplastic cells (Figure 1B) and in scattered large cells in the germinal center and in the subcapsular sinus of some lymph nodes (Figure 1C). The margins and tumor-free normal rectal mucosa did not show p16 expression.

**Figure 2:**
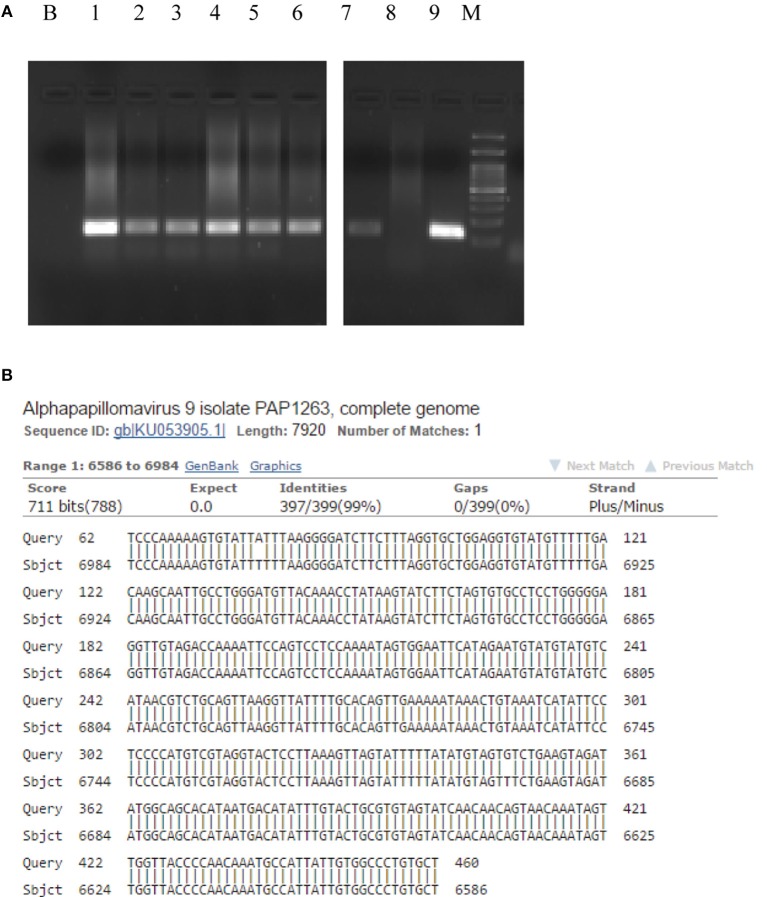
Human Papillomavirus polimerase chain reaction identification and typing. The presence of HPV 16 DNA was confirmed by HPV 16 genotype specific primer pair 5′- AAAGCCACTGTGTCCTGAAGA-3′ and 5′-CTGGGTTTCTCTACGTGTTCT-3′ able to amplify a 130 bps long fragment (424–553 nt, ref Seq Human Papillomavirus 16 type NC_001526). B, Blank, (1) Tumor mass, (2) Proximal margin, (3) Distal margin, (4) Lymph node, (5) Tumor-free mucosa of the surgical specimen, (6) Descending colon biopsies, (7) Caecum/ascending colon biopsies, (8) HPV DNA negative cervical cytological specimen, (9) HPV DNA positive cervical cytological specimen, M, Molecular weight marker **(A)**. HPV 16 was present in the tumor mass as it is evident by the sequence alignment of the 460 bps L1 sequence obtained by MY11/09 PCR primers **(B)**. HPV, Human Papillomavirus.

**Figure 3:**
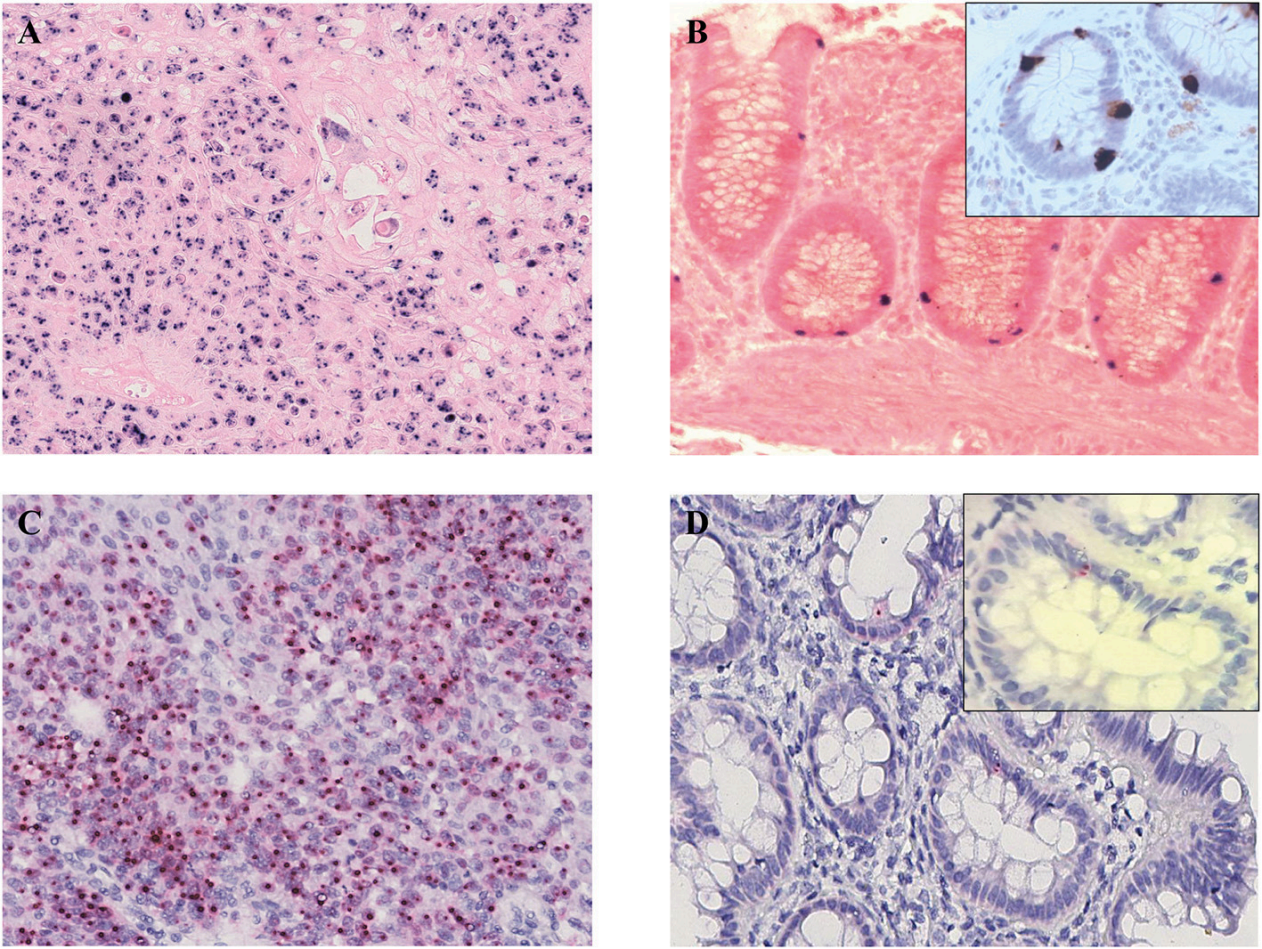
Human Papillomavirus detection and integration. In the neoplastic cells multiple black signals were shown by CISH with a “punctated” pattern characterized by multiple distinct dot-like intranuclear signals indicating viral integration **(A)**; the normal mucosa of the resected specimen demonstrated scattered cells with HPV infection in a “diffuse” pattern with only few completely black stained nuclei corresponding to the episomal status of the virus **(B)**. RNAscope assay detected *E6* and *E7* transcripts in the neoplastic **(C)** and normal mucosa cells of the surgical specimen (**D**, D inset) as multiple, strong red dot-like signals in the tumor and only few weak red signals in the cancer-free mucosa, corresponding the first pattern to viral integration and the second one to episomal infection. HPV-infected cells OCT3/4-positive (B inset). **(A,B)** CISH; **(C,D)** RNAscope; **(A,C)** original magnification (O.M.) 20x; **(B,D)** O.M. 40x; **(B,D)** inset, O.M. 63x. CISH, chromogenic *in situ* hybridization; HPV, Human Papillomavirus.

PCR assays for HPV DNA detection were performed by MY09/11 (17) primer pool, able to amplify about forty mucosal HPV DNAs, then with different primer set, including E1, E6, and L1 primers (Table 2). HPV DNA was found in the tumor, surgical margins, tumor free normal rectal mucosa proximal to the neoplastic cells and in the largest dissected lymph node (Figure 2A, Table 1). Sequence analysis of PCR products sustained the specificity of the PCR results and identified HPV 16 genotype (Figure 2B). The presence of HPV DNA in the tumor, surgical margins and tumor free normal rectal mucosa proximal to the neoplastic cells was also confirmed by CISH (Figures 3A,B) and RNAscope assay (Figures 3C,D, D inset, Table 1). As far as CISH and RNA scope assays is concerned, intriguingly, we detected two different pattern of expression in tumor and cancer-free samples. In the neoplastic tissue we observed a “*punctated*” pattern with distinct multiple dot-like intranuclear signals by CISH (Figure 3A) and strong red dot-like signals by RNAscope (Figure 3C), which are typical of viral integration. Whereas, in the non-neoplastic tissue we detected a “*diffuse*” black pattern by CISH (Figure 3B) and only few weak red signals by RNAscope (Figure 3D, D inset), which are typical of episomal DNA (18, 19). Therefore, the virus was integrated only in neoplastic cells.

Interestingly, in the non-neoplastic tissue, the HPV16 positive signals by CISH and RNAscope were localized within the gland crypts (Figures 3B,D). To better characterize the nature of these cells (endocrine vs. stem-cell), double staining for HPV (by CISH) and chromogranin, synaptophyn, OCT3/4 (by IHC) was performed. The infected cells were OCT3/4-positive and chromogranin/synaptophyn-negative, thus presumably representing stem-cells (Figure 3B, B inset, Table 3).

The expression of tumor suppressor genes p53, p21, and Rb was absent in the tumor mass and in adjacent tissues, thus supporting the view that HPV was oncogenic in our case.

At the first follow-up, multiple biopsies of caecum/ascending, and descending colon, as well as oral swab and anal and uretral scrapes were collected and analyzed for HPV infection (Table 1). HPV was no detected in the oral and ano-genital regions; we demonstrated the presence of HPV DNA in all of the biopsies of right and left colon by PCR (Figure 2A), whereas CISH and RNAscope assays were negative, thus confirming that the virus was integrated only in the neoplastic cells.

### HPV Circulation

**Figure 4:**
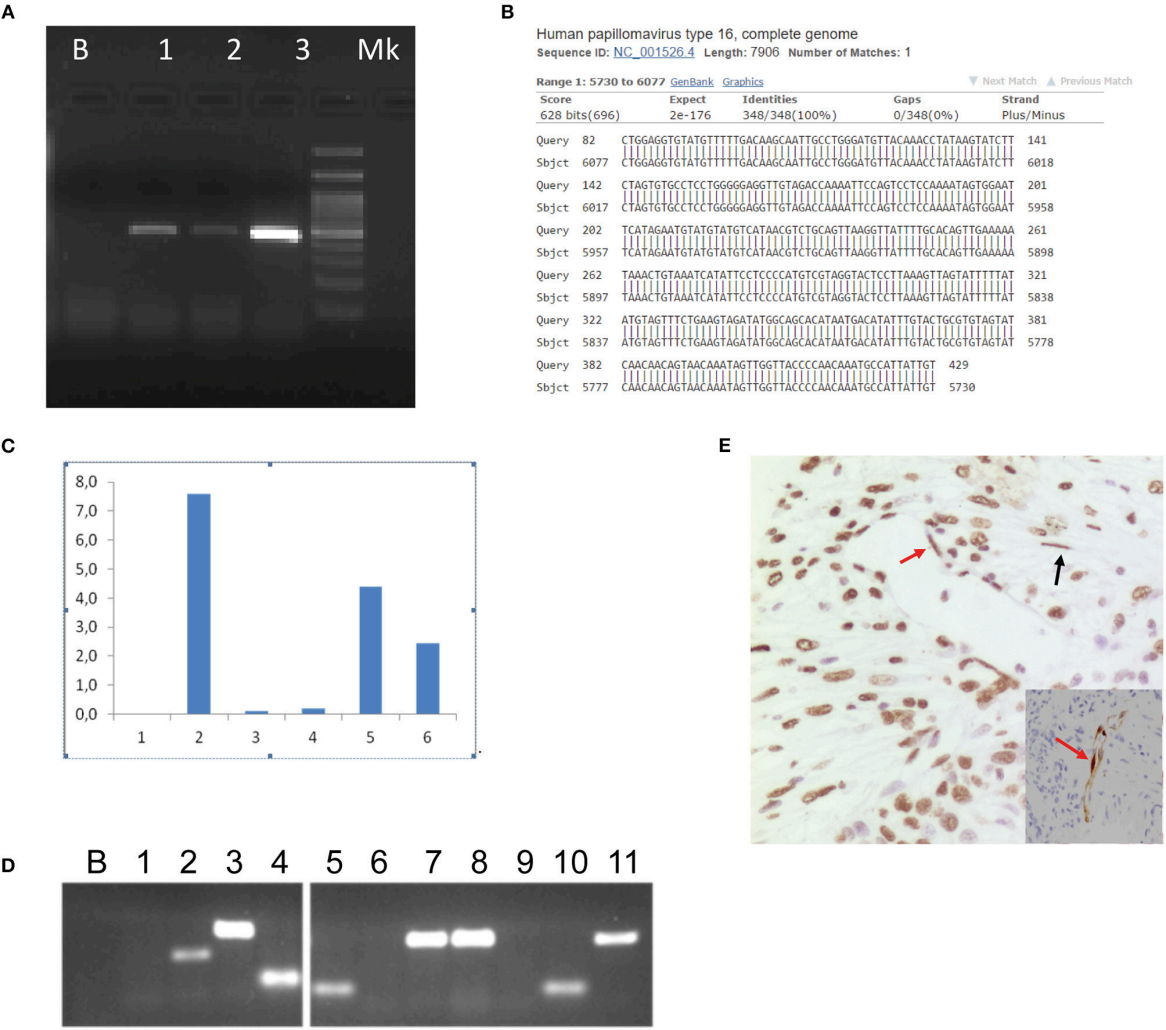
Human papillomavirus circulation. Electrophoresis showed the amplified HPV DNA obtained from plasma and Caski cells supernatant-derived exosomes. B, Blank; (1) Plasma-derived exosomes; (2) Caski cells; (3) Positive control (cervical HPV DNA positive cytological samples); M, Marker. The PCR assay was performed with MY11/09 primers **(A)**. Nucleotide blast of HPV DNA amplified product obtained from plasma-derived exosomes by MY11/09 primers **(B)**. HCT116 and NCM460 cell lines were exposed to CasKi supernatant derived exosomes and analyzed by Digital PCR (QuantStudio® 3D Digital PCR System, Life Technologies) to determine the capability of exosomes to transfer viral genetic material to recipient cells. Caski cell line was established from a metastasis in the small bowel mesentery and contains HPV16 DNA integrated into the genome. The reaction was performed using TaqMan Assay specific for HPV 16 E1 (Vi03453396_s1, Invitrogen, Milan, Italy). After 6 h of exposure the load of HPV DNA was greater than at 20 h in the normal human colon epithelial cell line (NCM460). In the human colon cancer cell line (HCT116) we report an HPV DNA burst at 6 h and a rapid decay, indeed it was absent at 20 h post exposure. (1) HCT116 at 3 h; (2) HCT116 at 6 h; (3) HCT116 at 20 h; (4) NCM460 at 3 h; (5) NCM460 at 6 h; (6) NCM4 60 at 20 h **(C)**. The specimens illustrated in **(C)** were also amplified by conventional PCR with E6 HPV 16 specific primer pair showing concordant results with Digital PCR. B, Blank, (1) not exposed HCT116; (2) HCT116 at 3 h; (3) HCT116 at 6 h; (4) HCT116 at 20 h; (5) not exposed NCM460; (6) NCM460 at 3 h; (7) NCM460 at 6 h; (8) NCM460 at 20 h; (9) blank; (10) HPV DNA negative cervical specimen; (11) HPV DNA positive cervical specimen **(D)**. The neoplastic and the neighbor cells demonstrated HPV DNA in the endothelial cells (red arrow) and in the fibroblasts (black arrow) by both CISH (brown chromogen) **(E)** and p16 IHC (**E**, inset). E, **(E)**-inset, original magnification 20x.

We searched for presence of HPV DNA in the plasma-derived exosomes of the patient (Figure 4A). Exosomes were isolated from plasma specimens by CD9 immunobeads isolation kit (HansaBioMed Life Sciences Ltd. Cod. HBM-BOLC-CC/20-1) according to manufacturer's instruction. Prior to DNA extraction of the exosomes content, we performed a DNAseI digestion to remove contaminant DNA. We searched for HPV DNA by using PCR assay (MY09/11 and E6 primers) and we found the presence of HPV 16 DNA, as resulted by sequence alignment of the PCR product (Figure 4B). Thus, we tested the hypothesis that HPV DNA could be transferred to recipient cells by exposing cells to HPV DNA positive exosomes. HPV DNA positive exosomes were obtained from the supernatant of an HPV DNA positive cell line, the Caski cell line, which was established from a metastasis in the small bowel mesentery and contains HPV16 DNA integrated into the genome. We confirmed *in vitro* the capability of HPV DNA positive exosomes to transfer their content to HPV DNA negative cells, such as neoplastic and normal colon cell lines, HCT116 and NCM460, respectively, by digital PCR and conventional PCR (Figures 4C,D). The amplification reaction was performed by Digital PCR, with TaqMan assay (Vi03453396_s1, Invitrogen, Milan, Italy) for HPV 16 E1 gene amplification and conventional PCR with E6 primers (Table 1). Interestingly, by CISH we found the presence of HPV DNA in the stromal cells (fibroblasts and endothelial cells) nearby the neoplastic cells, suggesting an horizontal transfer of viral DNA from cancer cells (Figure 4E).

### Microenvironment Characterization

**Figure 5:**
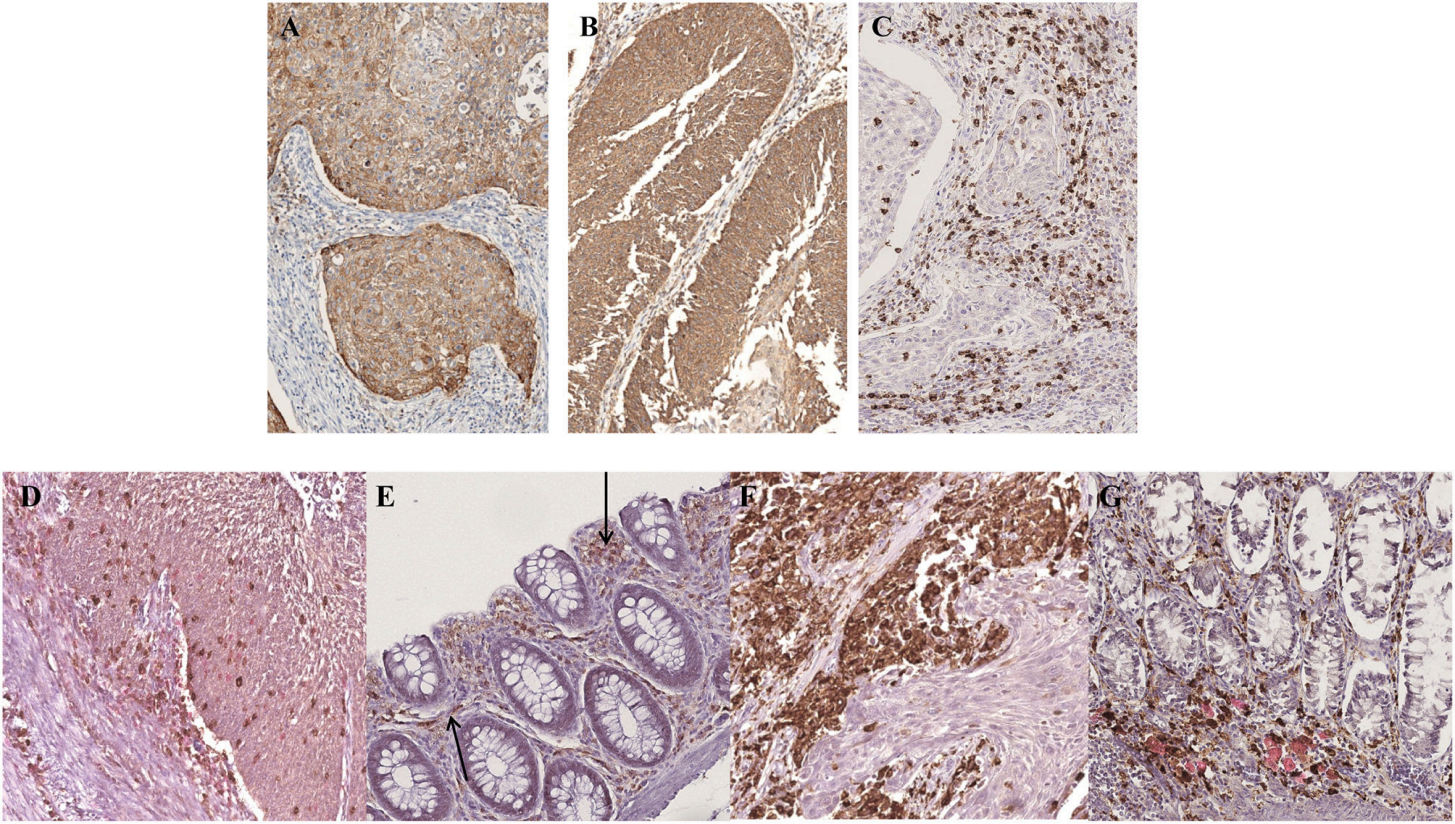
Microenvironment characterization. An increase of PDL-1 and PDL-2 expression in neoplastic cells was observed **(A,B)** whereas the reactive background showed a higher percentage of PD-1 positive elements **(C)**. The tumor infiltrating lymphocytes were represented mainly by CD8/PD-1 positive T-cells **(D)** which were absent in normal mucosa **(E)**. An increase of CD163/CD68-positive macrophages ratio was detected in the neoplastic component **(F)** as compared to the normal one **(G)**. **(A)** PDL-1 staining; **(B)** PDL-2 staining; **(C)** PD-1 staining; **(D,E)** CD8/PD-1 double staining (CD8 brown, PD-1 red); **(F,G)** CD163/CD68 double staining (CD163 brown, CD68 red). **(A–G)**, original magnification 20x.

More recently, local immune evasion has been involved in the development of gut cancer (20–22). Thus, we studied the neoplastic and non-neoplastic microenvironment of our patient by single and multiple IHC stainings. Interestingly, we demonstrated an increase of PDL-1 and PDL-2 expression in neoplastic cells coupled by PD-1 positivity of the reactive cells in the background (Figures 5A–C). This finding reflects a neoplastic environment with a reduced immune surveillance; in fact, the PD-1/PD-L1 molecular pathway can act to fine-tune the cellular fate of tumor-infiltrating T-cells. Interestingly, we detected an increase of T-regulatory cells (CD25/CD4/FOXP-3 positive) and a number of exhausted T-lymphocytes (CD8/PD-1 positive) in the neoplastic microenvironment when compared to non-neoplastic mucosa (Figures 5D,E). In addition, polarization of tumor-associated macrophages to an M2 phenotype was observed. In fact, the CD163/CD68 ratio was significantly higher in tumor than in non-neoplastic tissue (Figures 5F,G) (23) (Table 3).

## Discussion

Previous studies on HPV in CRC are conflicting (24). While some of them report HPV DNA in 31 −53% of CRC, others identified no HPV DNA (25, 26). Moreover, some Authors recognized transcriptional activity of the HPV *E6/E7* oncogenes critical to HPV's role in carcinogenesis, while others not (27–29). In our case study, HPV nucleic acids were detected in the neoplastic cells and in the normal crypts of the margins, non-neoplastic mucosa, random biopsies of the left and right colon as well as in some lymph nodes. It is known that inactivation of the *E2* gene through genomic integration promotes the expression of the E6 and E7 oncoproteins, which induce a loss of cell-cycle control by antagonizing the function of p53 and Rb, respectively (30). The resulting degradation of p53 and Rb mediated by these oncoproteins facilitates viral DNA proliferation within the host and leads to neoplasia (31). Accordingly, in our case we detected viral integration by p16 IHC, CISH, and RNA ISH for E6/E7 mRNA only in neoplastic cells coupled with no expression of p53, p21, and Rb. This supports the view that HPV might be involved in tumorigenesis, rather than simply representing a fortuitous or chance passenger through tissues. Interestingly, we did not find the virus in its physiologic ports of entry (i.e., oral and ano-genital region). This, along with the wide anatomical distribution of HPV in our samples implies that the infection was not just the result of a retrograde viral transmission from the ano-genital area. However, we cannot exclude that a past productive infection could has been the primary source of the widespread infection in our patient.

Previous studies on cervical, oropharingeal, and breast diseases demonstrated the presence of circulating HPV DNA in the blood of affected patients (8, 32–35). Accordingly, we searched HPV DNA also in the blood of our patient. Intriguingly, it was present in the CD9-positive fraction of the plasma, namely the exosomes fraction. Then, we demonstrated by *in vitro* studies that HPV DNA positive exosomes are capable to transfer the viral nucleic acids to HPV DNA negative cells, such as normal and transformed colon cell lines. *In vivo*, we observed a horizontal transfer of HPV DNA by a cell to cell communication mechanism to stromal cells (endothelial cells and fibroblasts) as well as at distant sites (ascending/descending colon). It is reasonable that HPV penetrated in the host by one of the known site of entry. Following, the virus disappeared from the original site thanks to clearing mechanisms (that are highly active during the early phase of HPV infection) and it was no more detectable there (2). HPV entered the blood circulation via exosomes with shedding of nucleic acids in target tissues (7–16). It is conceivable that the virus has elicited its oncogenic effect (as demonstrated by the lack of p53, p21, and Rb expression by IHC) specifically in the middle rectum for a temporary condition of immune evasion (36) characterized by up-regulation of T-regulatory and T-exhausted lymphocytes and a polarization of tumor-associated macrophages with a switching from M1 to M2 (17–19, 37) as demonstrated by IHC. Moreover, we showed high expression of PDL-1 and PDL-2 by neoplastic cells and strong positivity of the reactive background to PD-1. It is also possible that the virus by itself induces an immunomodulation as previously demonstrated for other viruses (37, 38). Notably, we identified the reservoir of HPV in non-neoplastic epithelial cells within the crypts of colonic normal mucosa adjacent to the neoplastic mucosa and we demonstrated that these cells were stem-cells, which are known to be susceptible to transformation by HPV 16 (39, 40). It can be argued that following inflammatory response to a number of stimuli (also including dysbiosis) (41), non-cancer stem cells may be activated into cancer stem cells, sustaining a feed-forward circuit of self-renewal, proliferation, differentiation and neoplastic transformation in a condition of immune evasion, as previously demonstrated (40, 42–44). Due to the pluripotency of stem-cells, a squamous cell carcinoma may arise also in colorectal mucosa in the absence of any metaplastic changes, as in our case.

## Concluding Remarks

Although important studies have been made for the understanding of HPV tumorigenicity in non-genital cancers, significant questions still remain. Our case-study seems to highlight the direct relationship of HPV with the pathogenesis of a squamous cell CRC; however, further studies including case control and cohort studies are necessary to evidence the causal link between HPV infection and colon cancer. The presence of HPV DNA positive plasma-derived exosomes in our patient shed important light on poorly understood mechanisms of HPV DNA horizontal transfer and on unconventional route of HPV spreading and carcinogenesis. It is noteworthy that HPV-induced cancerogenesis has a long-lasting history and our findings depicted only a static picture of a dynamic process for which we cannot exclude the development of cancers in other anatomic sites of the patient after viral spreading and DNA integration.

## Author Contributions

MRA, MC, and SL conceived and developed the study. LM, SDC, AC, AG, and BJR performed experiments. MC, MRA, SL, and SDC analyzed data. RV, ALF, TC, and VN collected and provided patient samples. MC, SL, MRA, and PT wrote the paper. PT provided his experience in fruitful discussion. All the authors discussed the results and commented on the manuscript.

### Conflict of Interest Statement

The authors declare that the research was conducted in the absence of any commercial or financial relationships that could be construed as a potential conflict of interest.
